# Bibliographical

**Published:** 1857-07

**Authors:** 


### BIBLIOGRAPHICAL.

We have received from the publishers Messrs. Blanchard & Lea, "The Physiological Anatomy and Physiology of Man." By Robert Bentley Todd, M. D., F. R. S., &c., and William Bowman F. R. S., &c., &c., Late Professors of Physiology and General Morbid Anatomy, in Kings College, London. Complete in one volume, with two hundred and ninety-eight illustrations. This is the conclusion of a work commenced in the year 1843, and intended as a text book for the Lectures on General Anatomy and Physiology, given in Kings College, London. The term Physiological Anatomy, has been adopted in preference to the older one of General, or the later one of Histological, as more comprehensive, and as intended to designate that kind of Anatomy, a knowledge of which, is especially required for practical investigations connected with physiological science.

In this work the Authors propose to give such a view of the main facts and doctrines of Anatomy and Physiology, particularly of those bearing on Practical Medicine and Surgery, as might suffice for the wants of the Student and Practitioner. They remark that, "Following that great master Haller, we were desirous of giving to Anatomy a greater degree of prominence, than had been usual in Physiological works, under the conviction that a thorough training in its several branches, descriptive, physiological, and comparative, is necessary to the formation of those habits of mind, which best fit the possessor for the successful investigation and the correct appreciation of physiological science."

We can, with the most entire confidence, recommend the work as having come up fully to the design of the Authors, and desire to express our pleasure in the fact, that so valuable a contribution to that department of Medical Science, (to the study of which our particular attention has for some time been directed) has been finally completed.

We next find upon our table A New American Edition (revised by the Author) of the Diseases of Women, including those of Pregnancy and Children. By Fleetwood Churchill, M. D., &c., &c., with notes and additions, by D. Francis Condie, M. D., &c.

It would seem almost useless for us to say anything in commendation of a work which has already established so enviable a reputation. Several new chapters have been added, and also a number of wood-cuts, and we have no hesitation in endorsing the statement, "that it is altogether a complete and faithful exponent of the present state of medical opinions and experience, in reference to the Pathology and Therapeutics of the entire range of diseases, to which the female sex is liable, including those of pregnancy and children."

We have also received a Manual of Examinations upon Anatomy, Physiology, Surgery, Practice of Medicine, Chemistry, Obstetrics, Materia Medica, Pharmacy and Therapeutics, especially designed for Students of Medicine, to which is added a Medical Formulary, by J. L. Ludlow, A. M., M. D. This is a new edition, thoroughly revised and much enlarged, with three hundred and seventy illustrations.

Since we must have works of this description, without giving any opinion as to the *real value* of such publications, our opinion is, that it is one of the best of its class, and to those for whom it was especially prepared, we recommend it as well adapted to the purpose for which it was designed.

Besides the books, we have received a number of pamphlets, which will be noticed upon some future occasion.

